# Procalcitonin guidance for reduction of antibiotic use in patients hospitalized with severe acute exacerbations of asthma: a randomized controlled study with 12-month follow-up

**DOI:** 10.1186/s13054-014-0471-7

**Published:** 2014-09-05

**Authors:** Wei Long, Li-juan Li, Gao-zhong Huang, Xue-min Zhang, Yi-cui Zhang, Jian-guo Tang, Yu Zhang, Gang Lu

**Affiliations:** Department of Internal and Geriatric Medicine, Shanghai Jiao Tong University Affiliated Sixth People’s Hospital, 222 Huan Hu Xi San Road, Shanghai, 201306 China; Department of Trauma-Emergency-Critical Care Medicine, Fudan University Affiliated Shanghai Fifth People’s Hospital, 128 Rui Li Road, Shanghai, 200240 China

## Abstract

**Introduction:**

Patients with severe acute exacerbations of asthma often receive inappropriate antibiotic treatment. We aimed to determine whether serum procalcitonin (PCT) levels can effectively and safely reduce antibiotic exposure in patients experiencing exacerbations of asthma.

**Methods:**

In this randomized controlled trial, a total of 216 patients requiring hospitalization for severe acute exacerbations of asthma were screened for eligibility to participate and 169 completed the 12-month follow-up visit. Patients were randomized to either PCT-guided (PCT group) or standard (control group) antimicrobial therapy. In the control group, patients received antibiotics according to the attending physician’s discretion; in the PCT group, patients received antibiotics according to an algorithm based on serum PCT levels. The primary end point was antibiotic exposure; secondary end points were clinical recovery, length of hospital stay, clinical and laboratory parameters, spirometry, number of asthma exacerbations, emergency room visits, hospitalizations and need for corticosteroid use due to asthma.

**Results:**

PCT guidance reduced antibiotic prescription (48.9% versus 87.8%, respectively; *P* < 0.001) and antibiotic exposure (relative risk, 0.56; 95% confidence interval, 0.44 to 0.70; *P* < 0.001) compared to standard therapy. There were no significant differences in clinical recovery, length of hospital stay or clinical, laboratory and spirometry outcomes in both groups. Number of asthma exacerbations, emergency room visits, hospitalizations and need for corticosteroid use due to asthma were similar during the 12-month follow-up period.

**Conclusion:**

A PCT-guided strategy allows antibiotic exposure to be reduced in patients with severe acute exacerbation of asthma without apparent harm.

**Trial registration:**

Chinese Clinical Trial Register ChiCTR-TRC-12002534 (registered 26 September 2012)

## Introduction

Asthma is a problem worldwide, with an estimated incidence of 300 million affected individuals and 250,000 annual deaths from asthma [1]. Acute exacerbations of asthma account for nearly 2 million emergency department (ED) visits and 500,000 admissions each year in the United States, frequently ranking as a major cause of absence from work and decreased productivity and incurring the greatest health-care costs [2,3]. Severe exacerbations of asthma are potentially life-threatening and often put patients at increased risk of ED admission and hospitalization.

Because most exacerbations of asthma are associated with viral respiratory tract infection (RTI) and bacterial infection seems to play only a minor role, global asthma management guidelines do not recommend routine use of antibiotics [4]. However, in countries with high prescription rates for antibiotics, many asthma patients with exacerbations are treated with antibiotics, leading to antibiotic overuse and bacterial resistance [5–7]. In the United States, approximately 22% of acute asthma patients in the ED receive an antibiotic unnecessarily [7]. In England, a high antibiotic prescription rate (57%) was observed in asthma patients [6]. In our previous study in China, about 70% of patients with mild to moderate acute exacerbation of asthma received antibiotics [5].

As many as 75% of all antibiotic doses are prescribed for RTIs, such as acute bronchitis, community-acquired pneumonia (CAP), acute exacerbations of asthma or chronic obstructive pulmonary disease (COPD), despite their mainly viral cause [8]. This inappropriate use of antibiotics is believed to be a main cause of the spread of bacterial resistance [9]. There is a need to reduce unnecessary antibiotic use during treatment of RTI, and much effort has been put into the search for sensitive and specific tools to guide antibiotic therapy in RTI patients. Clinical trials that have used procalcitonin (PCT) to guide antibiotic therapy for patients with RTI have shown that a biomarker-driven algorithm can cut antibiotic prescribing significantly and that this can be achieved without any increase in adverse events or treatment failures [5,10–17]. PCT-guided antibiotic stewardship reduced initial antibiotic prescription rates by 40% to 50% in patients with LRTI (lower respiratory tract infection) presenting to the ED [13] and by 70% to 80% in ambulatory patients presenting to their general physicians [15] and reduced total antibiotic exposure in CAP by 40% to 50% [10].

In our previous study, we demonstrated that PCT can be used accurately and effectively to determine whether acute asthma patients have bacterial infections and to guide the use of antibiotics in the treatment of acute exacerbation of mild to moderate asthma [5]. However, we included few severe asthma patients in the study, and the study had only a 6-week follow-up period. We therefore undertook a randomized controlled study to assess the usefulness of PCT guidance for antibiotic use in hospitalized patients with severe acute exacerbation of asthma, and this time included a 12-month follow-up period.

## Methods

### Patients and study design

This was a randomized controlled trial. The study was approved by the Shanghai Fifth People’s Hospital Research Ethics Committee, and all patients or their legally authorized representatives provided written informed consent.

From January 2009 to December 2011, consecutive patients ages 18 to 65 years who were hospitalized in the Fifth People’s Hospital of Shanghai with severe acute exacerbations of asthma were eligible for enrollment in the study. Asthma was defined according to the guidelines of the Global Initiative for Asthma [4]. A severe asthma exacerbation was defined as at least one of the following [18]: (1) need for systemic corticosteroids, or an increase from a stable maintenance dose, for at least 3 days and/or (2) hospitalization or ED visit because of asthma requiring systemic corticosteroids. Excluded were patients with antibiotic use within the previous 14 days, psychiatric disorders or other inability to give written informed consent, not being available for follow-up, severe immunosuppression, heart failure, cystic fibrosis, active tuberculosis, pregnancy and chest radiography–confirmed pneumonia.

Baseline assessment included asthma and medical history, vital signs, physical examination, routine blood tests, chest radiography, spirometry and clinical events related to asthma within the previous year, including asthma exacerbations, need for systemic corticosteroids, ED visits and hospitalizations for asthma. Spontaneously expectorated sputum samples were obtained to identify potential pathogenic microorganisms.

Serum PCT was measured using the B.R.A.H.M.S. PCT sensitive Kryptor (B.R.A.H.M.S., Hennigsdorf, Germany), a rapid sensitive assay with a functional sensitivity of 0.06 μg/L and a lower detection limit of 0.02 μg/L with an assay time of less than 30 minutes.

### Study intervention

Eligible patients were randomized to either PCT-guided (PCT group) or standard antimicrobial therapy (control group). Allocation to either intervention was conducted according to computer-generated random numbers produced by an independent statistician. After randomization, an opaque, sealed, sequentially numbered envelope containing the PCT or control protocol was prepared for each subject according to group assignment. The control group received antibiotic according to the discretion of the treating physician, who was unaware of the patient’s PCT levels. PCT levels of patients in the PCT group were provided to their attending physicians. Patients in the PCT group were treated with antibiotics on the basis of a PCT algorithm validated in previous studies [5,10,12–16]: antibiotic treatment was strongly discouraged when serum PCT level was less than 0.1 μg/L; antibiotic treatment was discouraged when serum PCT level was less than 0.25 μg/L; and antibiotic treatment was encouraged when serum PCT level was higher than 0.25 μg/L. When antibiotics were withheld from patients, a second measurement of the PCT level was mandatory within 6 to 24 hours for safety reasons. The use of antibiotics was recommended if this second measurement was higher than 0.25 μg/L. The physicians were permitted to overrule the algorithm, but they had to indicate the reasons for overruling. The patients were reevaluated 12 months after discharge.

### Outcome measures

The primary end point was antibiotic use, expressed as rate of antibiotic prescriptions in percentage and relative risk of antibiotic exposure. Secondary end points included measures of treatment success; length of hospital stay; clinical, laboratory and spirometry outcomes at discharge; and results of spirometry at the 12-month follow-up examination, as well as the results of the Asthma Control Test (ACT) [19], the results of the Asthma Quality of Life Questionnaire (AQLQ) [20] at the 12-month follow-up visit and the clinical events during the 12-month follow-up period, including numbers of asthma exacerbations, ED visits, hospitalizations and need for systemic corticosteroid use for treatment of asthma.

The ACT was used to determine the level of disease control. The overall ACT score varies from 5 (very poor asthma control) to 25 (full asthma control), with scores ranging from 20 to 24 defining controlled asthma, scores ranging from 16 to 19 partly indicating controlled asthma and scores ≤15 identifying uncontrolled asthma. The AQLQ was used to evaluate quality of life. The AQLQ consists of 32 items covering asthma-related symptoms and limitations during the 2 weeks preceding administration of the questionnaire, and responses are scored on a scale of 1 to 7, with higher numbers indicating a better quality of life. The validity and reliability of the ACT and the AQLQ have been evaluated for use in Chinese patients with asthma [21,22].

### Statistical analysis

Discrete variables are expressed as percentages and continuous variables as mean ± standard deviation (SD) for variables normally distributed and as median with interquartile range (IQR) for nonnormally distributed data. Comparability of groups was analyzed by χ^2^ test, two-sampled *t*-test and/or Mann-Whitney *U* test as appropriate. Relative risk (RR) was calculated with 95% confidence interval (CI). *P* < 0.05 was defined as statistically significant.

Assuming 90% of the patients in the control group would use antibiotics and anticipating a 15% decrease in antibiotic usage in the PCT group, a sample size of 158 patients (79 patients per group) was necessary to detect a significant difference in antibiotic prescription rate between the groups with 80% power and an α error of 0.05. To account for possible loss of patients to follow-up, we planned to enroll 180 patients.

The time to the first occurrence of asthma-related clinical events (exacerbation and hospitalization) was analyzed using Kaplan-Meier survival curves and by the logrank test. For outcomes during the follow-up period, we performed per-protocol analyses. Data were analyzed using SPSS version 16 for Windows statistical software (SPSS, Chicago, IL, USA).

## Results

### Study population

**Figure 1:**
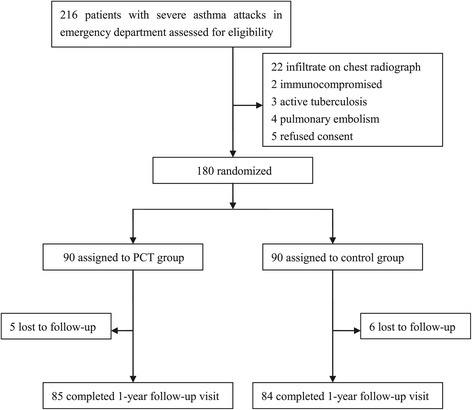
**Study flow diagram displaying the number of screened, excluded, randomized and analyzed patients.** PCT, Procalcitonin.

A total of 216 patients with severe acute exacerbations of asthma were screened for eligibility to participate in this study. Among this original sample, 180 were eligible and randomized into the PCT group (*n* = 90) or the control group (*n* = 90); of these 180 patients, 169 completed the 12-month follow-up visit, and 5 patients in the PCT group and 6 patients in the control group were lost to follow-up (Figure 1). The two treatment groups were matched at baseline for demographic, clinical and laboratory features (Table 1).

**Table 1:** **Characteristics of the study population at baseline** ^**a**^

**Characteristics**	**PCT group (** ***n*** **= 90)**	**Control group (** ***n*** **= 90)**	***P-*** **value**
Age in years, mean (SD)	40.4 ± 12.3	39.4 ± 11.7	0.594
Male sex, *n* (%)	49 (54.4)	52 (57.8)	0.652
Duration of asthma in years, mean (SD)	22.6 ± 11.7	21.8 ± 12.1	0.621
Smoking status, *n* (%)			
Current	17 (18.9)	14 (15.6)	0.553
Former	29 (32.2)	28 (31.1)	0.872
Never	44 (48.9)	48 (53.3)	0.551
Fever, *n* (%)	22 (24.4)	17 (18.9)	0.365
Cough, *n* (%)	69 (76.7)	63 (70.0)	0.312
Sputum, *n* (%)	56 (62.2)	51 (56.7)	0.447
Respiratory medication, *n* (%)			
ICS	23 (25.5)	27 (30.0)	0.505
ICS + LABA	59 (65.5)	56 (62.2)	0.641
HR, beats/min	101 ± 18	104 ± 16	0.319
RR, breaths/min	25 ± 7	24 ± 6	0.471
Systolic blood pressure, mmHg	111 ± 20	108 ± 19	0.271
pH	7.35 ± 0.08	7.36 ± 0.07	0.984
SaO_2_,%^b^	89 ± 5	88 ± 4	0.304
PaO_2_, mmHg^b^	61 ± 9	59 ± 8	0.178
PaCO_2_, mmHg^b^	58 ± 16	56 ± 14	0.410
CRP, mg/L	21.1 ± 9.1	22.3 ± 9.3	0.388
WBC, ×10^9^/L	11.8 ± 4.1	11.4 ± 3.7	0.598
PCT, μg/L	0.107 (0.052 to 0.273)	0.114 (0.055 to 0.266)	0.912
FEV_1_,% of predicted value	39.2 ± 9.6	39.8 ± 10.3	0.705
Clinical events related to asthma in previous year, *n* (%)			
Asthma exacerbations	78 (86.7)	75 (83.3)	0.531
Emergency room visits	69 (76.7)	65 (72.2)	0.494
Hospitalizations	17 (18.9)	14 (15.5)	0.553
Need for systemic corticosteroids for ≥3 days	53 (58.9)	49 (54.4)	0.547

^a^CRP, C-reactive protein; FEV_1_, Forced expiratory volume in 1 second; HR, Heart rate; ICS, Inhaled corticosteroid; LABA, Long-acting β_2_-agonists; PaCO_2_, Partial pressure of carbon dioxide; PaO_2_, Partial pressure of oxygen; PCT, Procalcitonin; RR, Respiratory rate; SaO_2_, Arterial oxygen saturation; WBC, White blood cells. ^b^Measured while patient was receiving oxygen therapy. Data are presented as number (%), median (IQR) or mean ± SD.

All patients received any one or a combination of the following medications for treatment of the exacerbation: theophylline, corticosteroids, β_2_-agonists and anticholinergic agents. There was a similar distribution of antibiotic use in both groups: macrolides (56.6%; PCT group, 32 cases; control group, 49 cases; *P* = 0.489), fluoroquinolones (23.1%; PCT group, 12 cases; control group, 21 cases; *P* = 0.924), aminopenicillin (11.2%; PCT group, 5 cases; control group, 11 cases; *P* = 0.609), cephalosporins (9.1%; PCT group, 4 cases; control group, 9 cases; *P* = 0.848). A single antibiotic was used in 108 patients (PCT group, 38 cases; control group, 70 cases; *P* = 0.715), and combination therapy with two or more antibiotics was administered in 15 patients (PCT group, 6 cases; control group, 9 cases; *P* = 0.715).

Sputum samples were obtained from 81 patients (PCT group, 39 cases; control group, 42 cases), and 24 causative microorganisms were isolated (PCT group, 11 organisms; control group, 13 organisms). The most frequently detected pathogens were *Chlamydophila pneumoniae* (PCT group, 4 organisms; control group, 6 organisms), followed by *rhinovirus* (PCT group, 1 organism; control group, 3 organisms) (Table 2).

**Table 2:** **Microbiology results from sputum in two groups** ^**a**^

	**Procalcitonin group (** ***n*** **= 90)**	**Control group (** ***n*** **= 90)**
*Influenza virus*, *n*	2	0
*Rhinovirus*, *n*	1	3
*Coronavirus*, *n*	1	1
*Chlamydophila pneumoniae*, *n*	4	6
*Mycoplasma pneumoniae*, *n*	1	0
*Pseudomonas aeruginosa*, *n*	1	0
*Streptococcus pneumoniae*, *n*	0	2
*Haemophilus influenzae*, *n*	1	1

The median PCT concentration was 0.113 μg/L (IQR, 0.054 to 0.270 μg/L), and 74 patients (41.1%) had PCT levels <0.1 μg/L, 30 (16.7%) had PCT levels between 0.1 and 0.25 μg/L and 76 (42.2%) had PCT levels >0.25 μg/L. PCT levels did not differ significantly between patients with sputum samples (0.106 μg/L; IQR, 0.050 to 0.266 μg/L) and those without (0.141 μg/L; IQR, 0.064 to 0.272 μg/L) (*P* = 0.145).

### Primary outcome

In the PCT group, 38 patients received antibiotics according to the PCT algorithm. Overruling was observed in six patients with PCT <0.25 μg/L. The reasons for overruling included receiving mechanical ventilation (*n* = 3), patient’s request (*n* = 1) and purulent sputum (*n* = 2). Four patients in the PCT group from whom antibiotics were initially withheld (those with a PCT level <0.25 μg/L) received an antibiotic based on a PCT level >0.25 μg/L at the second measurement during reevaluation after 6 to 24 hours. Overall, antibiotic exposure was lower in patients for whom antibiotics were prescribed according to the algorithm based on PCT levels compared to standard therapy (48.9% vs. 87.8%, respectively; *P* < 0.001) (RR, 0.56; 95% CI, 0.44 to 0.70; *P* < 0.0001). Thus, in spite of overruling, the PCT algorithm permitted a 44.3% reduction in antibiotic use.

### Secondary outcome

The rate of clinical success was comparable between the two groups. Fourteen patients (15.5%) in the PCT group and eleven (12.2%) in the control group received mechanical ventilation treatment (*P* = 0.518). The median of antibiotic duration in the PCT group (6 days; IQR, 4 to 9 days) was similar to that in the control group (6 days; IQR, 3 to 8 days) (*P* = 0.198). There were no significant differences between groups in laboratory measures, length of hospital stay or spirometry at discharge (Table 3).

**Table 3:** **Patient characteristics and outcomes at discharge** ^**a**^

	**PCT group (** ***n*** **= 90)**	**Control group (** ***n*** **= 90)**	***P***-**value**
Antibiotic use, *n* (%)	44 (48.9)	79 (87.8)	<0.001
Clinical success, *n* (%)	90 (100)	90 (100)	1.00
Duration of antibiotic, days	6 (4 to 9)	6 (3 to 8)	0.198
Length of hospital stay, days	9 (6 to 11)	8 (6 to 12)	0.380
Need for intubation, *n* (%)	14 (15.5)	11 (12.2)	0.518
FEV_1_,% of predicted value			
Prebronchodilator	70.9 ± 12.8	72.4 ± 11.2	0.404
Postbronchodilator	75.4 ± 11.6	77.1 ± 12.5	0.345
CRP, mg/L	9.3 ± 3.4	9.7 ± 4.2	0.509
WBC, ×10^9^/L	7.1 ± 2.1	7.4 ± 1.9	0.469
PCT, μg/L	0.041 (0.028 to 0.063)	0.046 (0.032 to 0.071)	0.723

^a^CRP, C-reactive protein; FEV_1_, Forced expiratory volume in 1 second; PCT, procalcitonin; WBC, white blood cells. Data are presented as number (%), median (IQR) or mean ± SD.

**Figure 2:**
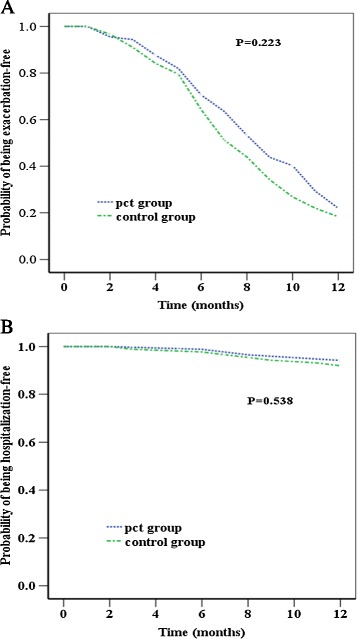
**Kaplan-Meier estimates of time to first asthma exacerbation (A) and hospitalization (B) after hospital discharge.** PCT, Procalcitonin.

Clinical and laboratory measures of outcome, spirometry and ACT and AQLQ scores were similar at the 12-month follow-up visit in both groups (Table 4). During the 12-month follow-up period, subsequent ED visits occurred in 119 cases (PCT group, 61 cases; control group, 58 cases; *P* = 0.698), and systemic corticosteroids for ≥3 days were needed in 89 cases (PCT group, 43 cases; control group, 46 cases; *P* = 0.587). There were no significant differences in the exacerbation rate (78.8% in PCT group vs. 82.1% in control group, respectively; *P* = 0.586) or in the hospitalization rate for exacerbations of asthma (8.2% in PCT group vs. 10.7% in control group, respectively; *P* = 0.582) (Table 3). The mean times to the first exacerbation and the first hospitalization due to exacerbation were also similar in both groups (Figure 2).

**Table 4:** **Comparison of patient characteristics and outcomes at the 12-month follow-up visit** ^**a**^

	**PCT group (** ***n*** **= 85)**	**Control group (** ***n*** **= 84)**	***P-*** **value**
Level of asthma control, *n* (%)			
Fully controlled	13 (15.3)	10 (11.9)	0.520
Controlled	48 (56.5)	51 (60.7)	0.575
Partly controlled	18 (21.2)	15 (17.8)	0.586
Uncontrolled	6 (7.1)	8 (9.5)	0.561
FEV_1_,% of predicted value			
Prebronchodilator	76.7 ± 11.8	77.9 ± 12.4	0.520
Postbronchodilator	83.5 ± 13.4	84.7 ± 12.7	0.551
ACT score, mean (SD)	21.1 ± 3.3	20.6 ± 3.5	0.324
AQLQ score, mean (SD)	5.04 ± 1.17	5.07 ± 1.21	0.788
Clinical events during 12-month follow-up period			
Asthma exacerbations, *n* (%)	67 (78.8)	69 (82.1)	0.586
Per patient per year	1.54 ± 1.17	1.52 ± 1.22	0.925
Emergency room visits, *n* (%)	61 (71.8)	58 (69.0)	0.698
Per patient per year	1.01 ± 0.81	0.96 ± 0.83	0.707
Hospitalizations, *n* (%)	7 (8.2)	9 (10.7)	0.582
Need for systemic corticosteroids for ≥3 days, *n* (%)	43 (50.6)	46 (54.8)	0.587

^a^ACT, Asthma Control Test; AQLQ, Asthma Quality of Life Questionnaire; FEV_1_, Forced expiratory volume in 1 second; PCT, Procalcitonin; Data are presented as number (%) or mean ± SD.

## Discussion

We observed a significant reduction in antibiotic use in patients hospitalized with severe acute exacerbations of asthma for whom a decision could be made on the basis of an algorithm of PCT measurements. Importantly, withholding antibiotic treatment did not cause apparent harm according to clinical and laboratory outcomes at discharge and during the 12-month follow-up period.

Exacerbation of asthma is often precipitated by a viral respiratory infection, and atypical bacteria such as *Mycoplasma pneumoniae* and *Chlamydophila pneumoniae* may play a role in its pathogenesis [23–25]. Antibiotic use during treatment of asthma exacerbation remains controversial, and few patients are estimated to benefit from this type of antibiotic treatment. In the TELICAST study [26], a total of 278 adults with acute exacerbation of asthma were randomly assigned to receive 10 days of treatment with oral telithromycin or placebo in addition to usual care. Patients in the telithromycin group had a significantly greater improvement in asthma symptoms, but they did not show an improvement in peak expiratory flow rates measured in the morning at home. However, routine use of antibiotics for exacerbations of asthma is of no benefit and may cause unwanted effects, so the empirical use of antibiotics is not recommended. In a randomized double-blind study [27], Graham and colleagues assessed the value of amoxicillin in 60 adults admitted to the hospital with acute exacerbations of asthma, with 37 exacerbations treated with amoxicillin and 34 with placebo. Response to treatment was closely monitored, but no significant differences between groups were demonstrated with regard to improvement measured by length of hospital stay, time until 50% improvement in symptoms, patient self-assessment and respiratory function, and symptoms and respiratory function at discharge. More recently, in the AZISAST study [28], 55 patients were randomly assigned to receive azithromycin and 54 were to receive placebo. Add-on treatment with azithromycin did not decrease the frequency of severe exacerbations of asthma and LRTI requiring antibiotics.

Under realistic clinical practice conditions, the challenge for physicians is to identify asthma patients with bacterial RTI for whom antimicrobial treatment is likely to be beneficial. Patients are often prescribed antibiotics despite clinical practice guideline recommendations against the empiric use of antibiotics in acute exacerbations of asthma [5–7]. Clinical signs and symptoms of acute asthma and bacterial RTI overlap, as do commonly used laboratory parameters such as C-reactive protein (CRP) and white blood cell (WBC) count, making it difficult for physicians to distinguish viral from bacterial infections in asthma patients [29,30]. Patients with severe exacerbations are more often treated with antibiotics because of high morbidity and mortality. The routine use of standard laboratory tests such as CRP level and WBC count seems to be motivated more by their low cost, easy availability and historic practice rather than by their diagnostic accuracy. Moreover, the reliability of CRP level and/or WBC count for guiding antimicrobial therapy is limited by their late peak levels as well as their suboptimal specificity, especially in patients with systemic inflammation [31,32].

Circulating levels of PCT are elevated in patients with systemic bacterial infections, but they remain relatively low in patients with viral infections or inflammatory diseases [33,34]. Hence, PCT levels may be used to support clinical decision-making for the initiation and discontinuation of antibiotic therapy [35]. Since the first pilot study published in 2004 [10], PCT-guided strategies for antibiotic treatment decision have gained much attention. Overall, it has been shown that PCT might be feasible in guiding antibiotic treatment in patients with acute exacerbations of COPD [12] as well as in limiting treatment duration in patients with CAP [11] and severe infectious diseases in the intensive care unit [36–39]. The main effect has been achieved either by discouraging antibiotic initiation or by shortening the duration of antibiotic treatment without increased rates of adverse outcomes [10–14,37]. The use of PCT also has prognostic implications, as falling values correlate with good outcomes and static or increasing values correlate with adverse outcomes, including mortality [40,41]. More recently, a PCT strategy was evaluated in primary care in patients with symptoms of acute respiratory infections, with results supporting the potential for substantial reductions in antibiotic use in this patient population [13,15].

Only a few asthma patients were included in two previous trials of PCT-guided therapy, [10,13]. In our own previous study, only 34 patients with severe exacerbation asthma were included [5]. The presently reported trial is, to our knowledge, the first clinical study focused on evaluation of the PCT test in its effectiveness in guiding antibiotic treatment of severe acute exacerbations of asthma.

However, some limitations of the present study should be recognized. First, we excluded patients older than 65 years of age, which may limit the application of our findings to older patients. Second, we did not conduct a formal cost–benefit study. To evaluate if a PCT-based strategy is cost-efficient under real-life conditions, cost aspects should be carefully considered. Third, this study was performed in a single center with a high baseline use of antibiotics for the treatment of asthma. Reductions in antibiotic use by means of PCT therapy guidance will be less in centers that already use antibiotics for asthma less often. Fourth, because of its relatively small sample size, our study has limited power to prove the safety of the PCT strategy, and we cannot exclude a potential harm of reducing antibiotic therapy on the basis of PCT guidance. Taking into account the limited population size in the study, a multicenter trial that includes a large number of patients with severe exacerbation of asthma is desirable to validate our data. We emphasize that any infection is too complex to be reduced to a single cutoff of a surrogate marker, and biomarkers should complement rather than replace good clinical practice. Certainly, results from the use of biomarkers should be considered in light of the pretest probability of disease presence.

## Conclusions

The results of our study support the concept that PCT guidance reduces antibiotic exposure in patients with severe acute exacerbation of asthma without apparent harm. Given the prevalence of asthma and the duration of illness, a reduction in antibiotic prescriptions for the treatment of exacerbations could result in fewer side effects, lower costs and, in the long term, lead to diminishing drug resistance, particularly in countries such as China, where patients are suffering from the overuse of antibiotics [42,43].

## Key messages

- Antibiotic overuse in China is normal, and patients with severe acute exacerbations of asthma often receive inappropriate antibiotic treatment.
- The results of this single-center study show that a significant reduction in antibiotic use in patients hospitalized with severe acute exacerbations of asthma for whom a decision could be made on the basis of an algorithm of PCT measurements.
- Withholding antibiotic treatment on the basis of PCT guidance did not cause apparent harm for up to 12 months.
- Additional larger multicenter studies are needed to confirm the usefulness of PCT guidance during treatment of severe asthma exacerbation.

## Abbreviations

ACT: Asthma Control Test
AQLQ: Asthma Quality of Life Questionnaire
CAP: Community-acquired pneumonia
CI: Confidence interval
COPD: Chronic obstructive pulmonary disease
CRP: C-reactive protein
ED: Emergency Department
ICS: Inhaled corticosteroid
IQR: Interquartile range
LABA: Long-acting β_2_-agonist
PCT: Procalcitonin
RR: Relative risk
RTI: Respiratory tract infection
SD: Standard deviation
WBC: White blood cell
